# Endometriosis-associated ovarian cancer: A ten-year cohort study of women living in the Estrie Region of Quebec, Canada

**DOI:** 10.1186/1757-2215-3-2

**Published:** 2010-01-19

**Authors:** Aziz Aris

**Affiliations:** 1 Department of Obstetrics and Gynecology, Sherbrooke University Hospital Centre, 3001, 12e Avenue Nord, Sherbrooke, Quebec, J1H 5N4 Canada

## Abstract

**Objectives:**

Endometriosis has been believed to increase the risk of developing ovarian cancer, but recent data supporting this hypothesis are lacking. The aim of this study was to verify whether the incidence of endometriosis, ovarian cancer and the both increased during the last 10 years among women living in the Estrie region of Quebec.

**Methods:**

We collected data of women diagnosed with endometriosis, ovarian cancer or both, between 1997 and 2006, from a population living in the Estrie region of Quebec. We performed this retrospective cross-sectional study from the CIRESSS (Centre Informatisé de Recherche Évaluative en Services et Soins de Santé) system, the database of the CHUS (Centre Hospitalier Universitaire of Sherbrooke), Sherbrooke, Canada.

**Results:**

Among the 2854 identified patients, 2521 had endometriosis, 292 patients had ovarian cancer and 41 patients had both ovarian cancer and endometriosis. We showed a constant increase in the number of ovarian cancer (OC) between 1997 and 2006 (r^2 ^= 0.557, P = 0.013), which is not the case for endometriosis (ENDO) or endometriosis-associated ovarian cancer (EAOC). The mean age ± SD was 40.0 ± 9.9 and 53.9 ± 11.4 for patients having ENDO and OC, respectively. Mean age of women with EAOC was 48.3 ± 10.8, suggesting an early onset of ovarian cancer in women having endometriosis of about 5.5 years average, P = 0.003. Women with ENDO were at increased risk for developing OC (Rate Ratio [RR] = 1.6; 95% Confidence Interval [CI] = 1.12-2.09). Pathological analyses showed the predominance of endometrioid type (24.4%) and clear-cell type (21.9%) types in EAOC compared to OC, P = 0.0070 and 0.0029, respectively. However, the serous type is the most widespread in OC (44.5%) in comparison to EAOC (19.51%), P = 0.0023.

**Conclusion:**

Our findings highlight that the number of cases of ovarian cancer is constantly increasing in the last ten years and that endometriosis represents a serious risk factor which accelerates its apparition by about 5.5 years.

## Background

According to Ovarian Cancer Canada citing Statistics from the National Ovarian Cancer Survey: Perspectives of Canadian Women and Health Care Professionals (1999) [1]. Ovarian cancer (OC) affects about 1 in 70 Canadian women. About 2300 new cases of ovarian cancer are found in women in Canada each year. About 1600 Canadian women die each year of this disease, making it the fifth ranking cause of cancer deaths. Six out of ten women diagnosed with ovarian cancer in Canada are 50 to 79 years of age [1]. The high mortality rate arises mainly because the disease is asymptomatic in its initial stages, making its early detection difficult [2]. At the time of diagnosis, dissemination has occurred in more than 70% of cases, at which point the 5-year survival rate is less than 20%. Ninety percent of all ovarian cancers are epithelial in origin, and are classified according to their cell types (serous, mucinous, endometrioid, clear cell and undifferentiated or mixed histology) [2].

Different etiological factors have been implicated in ovarian cancer types although, at present, little is known about the molecular events involved in their individual development. Some types such as clear-cell and endometrioid have been shown associated with the benign disease, endometriosis [3]. This disease is a complex genetic trait which affects up to 10% of women in their reproductive years [4]. It causes pelvic pain, severe dysmenorrhea (painful periods) and subfertility [5]. The disease is defined by the presence of endometrial-like epithelium and stroma in the extra-uterine sites, most commonly the ovaries and peritoneum. The main pathological processes associated with endometriosis are peritoneal inflammation and fibrosis, and the formation of adhesions and endometriomas (benign ovarian cysts). Circumstantial evidence that endometriosis is an endometriosis-associated ovarian cancer precursor has been accumulating over many years.

Although many of the risk factors associated with both diseases are similar, including earlier menarche, more regular periods, shorter cycle length and lower parity, endometriosis itself may be a risk factor for ovarian cancer. However, the results of observational studies of the association between these two diseases are inconsistent. Some studies showed an increased risk for ovarian cancer [6-10], while other studies did not confirm such an association [11-13]. The aim of this study was to clarify further the relationship between ovarian cancer and endometriosis in the Estrie region of Quebec, Canada.

## Materials and methods

Located at the CHUS (Sherbrooke University Hospital Centre), the CIRESSS (Centre Informatisé de Recherche Évaluative en Services et Soins de Santé) system manages clinical and pathological data obtained from the computerized patients' records of all residents in the Estrie region of Quebec. It covers 300 383 individuals, and it is principally based on clinical and pathological reports. Cancer incidence was coded according to the second edition of the International Classification of Diseases for Oncology (ICD-O-2). Endometriosis was coded according to the International Classification of Diseases, Ninth Revision, Clinical Modification [ICD-9-CM], codes 617.00-617.99.

This retrospective study is based on women with ovarian cancer, who were identified from the archive of the CIRESSS Registry during a 10-year period (1997-2006). A total of 2521 female patients with endometriosis were present, as well as 292 with ovarian cancer. A total of 41 women had ovarian cancer and endometriosis. Codes for ovarian cancer and endometriosis, respectively, were extracted from the archive, and the demographical and pathological data were analyzed. We reviewed medical and pathological reports to confirm the presence of ovarian cancer and/or endometriosis. Histological types of ovarian cancer were obtained for ovarian cancer associated or not associated with endometriosis.

The study was approved by the CHUS Ethics Human Research Committee on Clinical Research, the Research Ethics Board of the CHUS, and received the number of approbation # 07-054.

### *Statistical Analysis*

Analysis of variance (ANOVA) was used to evaluate if there were significant differences for age of patients between the studied groups. To compare the proportion of histological type between the groups with ovarian cancer or endometriosis-associated ovarian cancer, the test of Chi-Square was used or Fisher Exact test when frequency are smaller than 5.

It was interesting to study if the number of subjects increased in each of the groups by years. A simple linear regression was thus realized to compare the percentage of subjects by years with regard to the number of total subjects over the period studied according to years. One regression was realized by groups studied.

Analyses were realized with the software SPSS version 14.0 and the StatXact version 6.0. A value of p < 0.05 was considered as significant for every statistical analysis.

## Results

**Figure 1:**
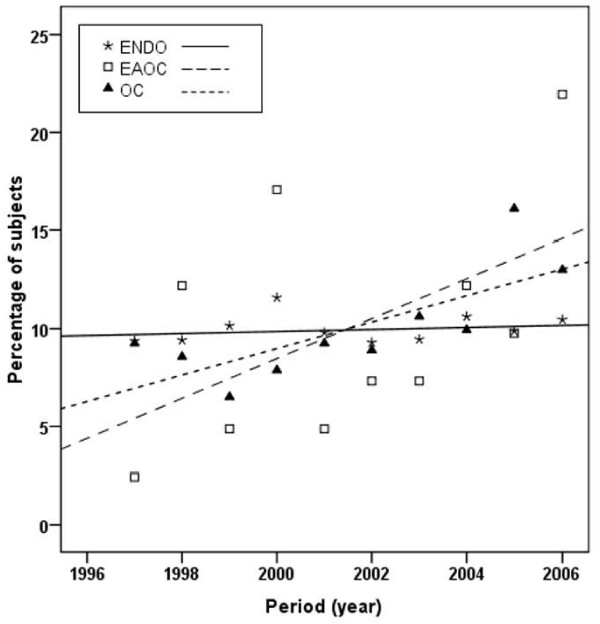
**Annual variation of the percentage of endometriosis (ENDO), endometriosis-associated ovarian cancer (EAOC) and ovarian cancer (OC) cases between 1997 and 2006**. Using simple linear regression analysis, there was no annual significant increase of cases of endometriosis **(ENDO: asterisk) **and endometriosis-associated ovarian cancer **(EAOC: white square)**, r^2 ^= 0.046, P = 0.550 and r^2 ^= 0.263, P = 0.123; respectively. However, the percentage of cases of ovarian cancer **(OC: black triangle) **was increased between 1997 and 2006, r^2 ^= 0.557, P = 0.013.

As shown in Table 1, among the 2854 identified patients, 2521 had endometriosis (ENDO), 292 patients had ovarian cancer (OC) and 41 patients had the both, i.e. endometriosis-associated ovarian cancer (EAOC). Using simple linear regression analyses (Figure 1), we showed that there is a constant increase in the number of OC between 1997 and 2006 (r^2 ^= 0.557, P = 0.013), whereas this increase has not been demonstrated in endometriosis alone (r^2 ^= 0.046, P = 0.550) or endometriosis-associated ovarian cancer (r^2 ^= 0.263, P = 0.123).

**Table 1:** Age at diagnosis of endometriosis (ENDO), ovarian cancer (OC) and endometriosis-associated ovarian cancer (EAOC) groups

	N	Age	SD
**ENDO**	2521	40.0**†**	9.9
**OC**	292	53.8**†**	11.4
**EAOC**	41	48.3**†**	10.8
**Total**	2854	41.6	10.9

**†**Significant difference between groups in the age at endometriosis or ovarian cancer diagnosis (P < 0.0001). After Tukey adjustment, mean difference (± SE) of age was 8.2 ± 1.6, P < 0.0001 between EAOC and ENDO; -5.5 ± 1.7, P = 0.003 between EAOC and OC; and -13.8 ± 0.6, P < 0.0001 between ENDO and OC.

After adjusting for age, number of pregnancies, family history of ovarian cancer, race, oral contraceptive use, tubal ligation, hysterectomy and breastfeeding; women with endometriosis were at increased risk for developing ovarian cancer (Rate Ratio [RR] = 1.6; 95% Confidence Interval [CI] = 1.12-2.09). According to census data from 2001 of the Estrie Region, prevalence was 10.7% and 0.11% for ENDO and EAOC, respectively; ENDO among OC cases was 14% and incidence of OC was 24% (Table 2). Of note, prevalence and incidence were compared between our results and literature [14-16] (Table 2).

**Table 2:** Prevalence and incidence of endometriosis (ENDO), ovarian cancer (OC) and endometriosis-associated ovarian cancer (EAOC), according to our data and literature

	Our data	Literature	References
**Prevalence of ENDO**	10.7%	10%	Wheeler et al. 1989
**Prevalence of EAOC**	0.11%	0.09%	Wheeler et al. 1989; Van Gorp et al. 2004
**Risk of malignant transformation in ENDO**	1.63%	0.7-4.5%	Kobayashi et al. 2009; Van Gorp et al. 2004
**ENDO among OC cases**	14.0%	-	-
**Incidence of OC**	24.2†	13.2	Kobayashi et al. 2009

†Incidence of OC = 24.2/100 000

The mean age ± SD was 40.0 ± 9.9 and 53.9 ± 11.4 for patients having endometriosis or ovarian cancer (OC), respectively. Mean age of women with endometriosis-associated ovarian cancer (EAOC) was 48.3 ± 10.8 (Table 1). Furthermore, data after Tukey adjustment showed that mean (± SE) difference of age at ovarian cancer diagnosis was 5.5 ± 1.7 between EAOC and OC, P = 0.003, suggesting an early onset of ovarian cancer in women having endometriosis of about 5.5 years average (Table 1). The mean difference of age was 8.2 ± 1.6 late in EAOC compared to ENDO, P < 0.0001; and 13.8 ± 0.6 earlier in ENDO compared to OC, P < 0.0001 (Table 1).

Pathological analyses, as summarized in Table 3, showed that 2 types of ovarian cancer predominate in EAOC in comparison to OC: endometrioid type (24.4%, P = 0.007) and clear-cell type (21.9%, P = 0.003), whereas serous type (44.5%) was predominant in OC compared to EAOC, P = 0.002. Difference in percentage between endometrioid, clear-cell and serous types in the EAOC group was not statistically significant. On the other hand, all other types (i.e. epidermoid, mixed, etc) represent 36.6% in EAOC and 38.4% in OC, without statistically significance.

**Table 3:** Groups of endometriosis-associated ovarian cancer (EAOC) and ovarian cancer (OC), according to histological types

	EAOC		OC		P value
	N	%	N	%	
**Clear-cell type**	9	21.95	22	7.53	0.0029*
**Endometrioid type**	10	24.39	29	9.93	0.0070*
**Mucinous type**	2	4.88	6	2.05	0.2571
**Serous type**	8	19.51	130	44.52	0.0023*
**Other types**	15	36.58	112	38.36	0.8270

Comparisons were performed between endometriosis-associated ovarian cancer (EAOC) and ovarian cancer (OC).

* P < 0.01 for clear-cell, endometrioid, and serous types.

## Discussion

Ovarian cancer belongs to the most lethal class of gynecological malignancies and remains the fifth most common cause of cancer-related deaths among women [17]. The overall 5-year survival rate remains poor, despite significant improvements in surgical treatment and chemotherapy. The first interesting results in our study were the constant increase of the number ovarian cancer since 1997 to 2006. This increase is not supported by literature [16,18] and should attract attention. Other studies should confirm if this increase is a geographically widespread or localized phenomenon. Further investigations should also be undertaken to find out more about the causes that have contributed to this increase.

The second findings of our study showed that the cohort of endometriosis women had an increased risk of ovarian cancer of 1.6 (95% CI 1.12-2.09). These data are consistent with other studies highlighting the increased risk of ovarian cancer in endometriosis patients [6,7,18]. Since endometriosis is a common benign gynecological disorder affecting 10% of women during their reproductive years [19], with a reported prevalence going as high as 15-25% in some studies [18,20], any association with OC is concerning. Prevalence and incidence were obtained using census data from 2001 of the Estrie Region (Census of Canada, 2001). Observed results are consistent with literature. However, we noticed an increased incidence of OC in our region compared to data from the US National Cancer Institute for the same period. Also, it is known that prevalence of ENDO (in our region as in literature) is probably an under-estimation since an unknown proportion of women suffering of ENDO do not consult for their symptoms.

On the other hand, the age at ovarian cancer diagnosis, in our study, is of about 54 years and this is consistent with previous studies [21,22]. It is interesting to note that the mean age at diagnosis of ovarian cancer in endometriosis patients is of 48 years, therefore earlier about 5.5 years average. This difference in the age at diagnosis might be explained by the fact that women with ENDO consult earlier owing to associated symptoms. However, the hypothesis of an earlier development of the OC in women with ENDO cannot be ruled out. The earlier manifestation of OC has serious consequences, since survival in cases of ovarian cancer rarely exceeds 5 years.

Our results showed that 3 types of ovarian cancer are more common in patients with endometriosis compared to those without: endometrioid type (24.4%) and clear-cell type (21.9%). Our findings are consistent with other studies supporting the predominance of endometrioid and clear-cell types of ovarian cancer-associated endometriosis [18,23,24].

## Conclusion

Our study reveals that the number of cases of OC has increased steadily over the last decade in our region and that endometriosis represents a risk factor associated with an earlier time of diagnosis of about 5.5 years in relation to its apparition. On the other hand, despite the pathophysiological and epidemiological evidence linking endometriosis with ovarian cancer, it is still unclear whether endometriosis is a precursor to EAOC, or whether there is an indirect link involving common environmental, immunological, hormonal or genetic factors. Further investigations are needed to provide answers to these pertinent questions.

## List of abbreviations

ENDO: endometriosis; OC: ovarian cancer; EAOC: endometriosis-associated ovarian cancer; RR: rate ratio; CI: confidence interval.

## Competing interests

The author declares that they have no competing interests.

## Authors' contributions

AA: Professor, responsible for the project. He was involved in all steps of the work (i.e. conception, design, analysis and interpretation of data, and drafting the manuscript).
